# Supplementary material for: Differential Expression of HOX Genes in Mesenchymal Stem Cells from Osteoarthritic Patients Is Independent of Their Promoter Methylation
Source: Cells. 2018 Dec 5;7(12):244. doi: 10.3390/cells7120244 (PMC6316585; doi:10.3390/cells7120244)

Supplementary file Table S1

Grouping of Homeobox genes according to their main known function.

Anatomical Structure Morphogenesis	*EN1*, ***HOXC10***, ***HOXC13***, ***HOXD3***, *LBX1*, *SIX2*, *SIX4*
Organ Morphogenesis	*CDX1*, *CDX2*, ***HOXA11***, ***HOXA13***, *ISL1*, *LHX1*, *PAX3*, *PDHX*, *PITX2*, *PITX3*, *PROX1*, *SIX6*
Body Pattern Formation	*ALX3*, *EMX2*, *HHEX*, ***HOXA11***, ***HOXA2***, ***HOXA4***, ***HOXA5***, ***HOXA6***, ***HOXB1***, ***HOXB5***, ***HOXB6***, ***HOXC5***, ***HOXD10***, ***HOXD8***, *LMX1B*, *PITX2*
Ectoderm Development	*PROX1*, *VAX2*
Endoderm Development	***HOXC11***
**Brain & Nervous System Development**	
Brain Development	*ALX1*, *DLX2*, *EMX2*
Nervous System Development:	*ARX*, *DLX5*, *DLX6*, ***HOXD10***, *LBX1*, *LHX1*, *OTP*, *PAX3*, *PHOX2A*, *PHOX2B*
**Skeletal Development:**	*ALX3*, *ALX4*, *DLX3*, *DLX5*, *DLX6*, *EN1*, ***HOXA11***, ***HOXA13***, ***HOXA2***, ***HOXB6***, ***HOXD10***, ***HOXD13***, *MSX2*
**Muscle Development:**	*BARX2*, *MKX*, *SIRT1*, *SIRT2*, *SIX1*
**Other Homeobox Genes Involved In Multicellular Organismal Development:**	*BARX1*, *CDX4*, *CUX1*, *DLX1*, *EMX1*, *EN2*, ***HOXA1***, ***HOXA7***, ***HOXA9***, ***HOXB13***, ***HOXB2***, ***HOXB3***, ***HOXB4***, ***HOXB7***, ***HOXB8***, ***HOXB9***, ***HOXC12***, ***HOXC8***, ***HOXC9***, ***HOXD1***, ***HOXD11***, ***HOXD12***, ***HOXD9***, *ISL2*, *LBX2*, *LMX1A*, *MEIS1*, *NKX3-1*, *OTX1*, *TLX1*, *VAX1*, *VSX1*, *VSX2*
**Homeobox Genes Involved In Cell Differentiation:**	*ARX*, *EMX2*, *HHEX*, *HLX*, *HOPX*, *LBX1*, *LHX1*, *LMX1B*, *MIXL1*, *OTP*, *PHOX2A*, *SIRT1*, *VSX2*
**Other Genes:**	*PHTF1*, *SIRT3*, *SIRT6*, *SIRT7*, *ZHX1*, *ZHX2*

Homeobox genes include two subsets of genes coding for transcription factors involved in multiple functions. The clustered HOX genes are indicated in bold.

Supplementary file Figure S2

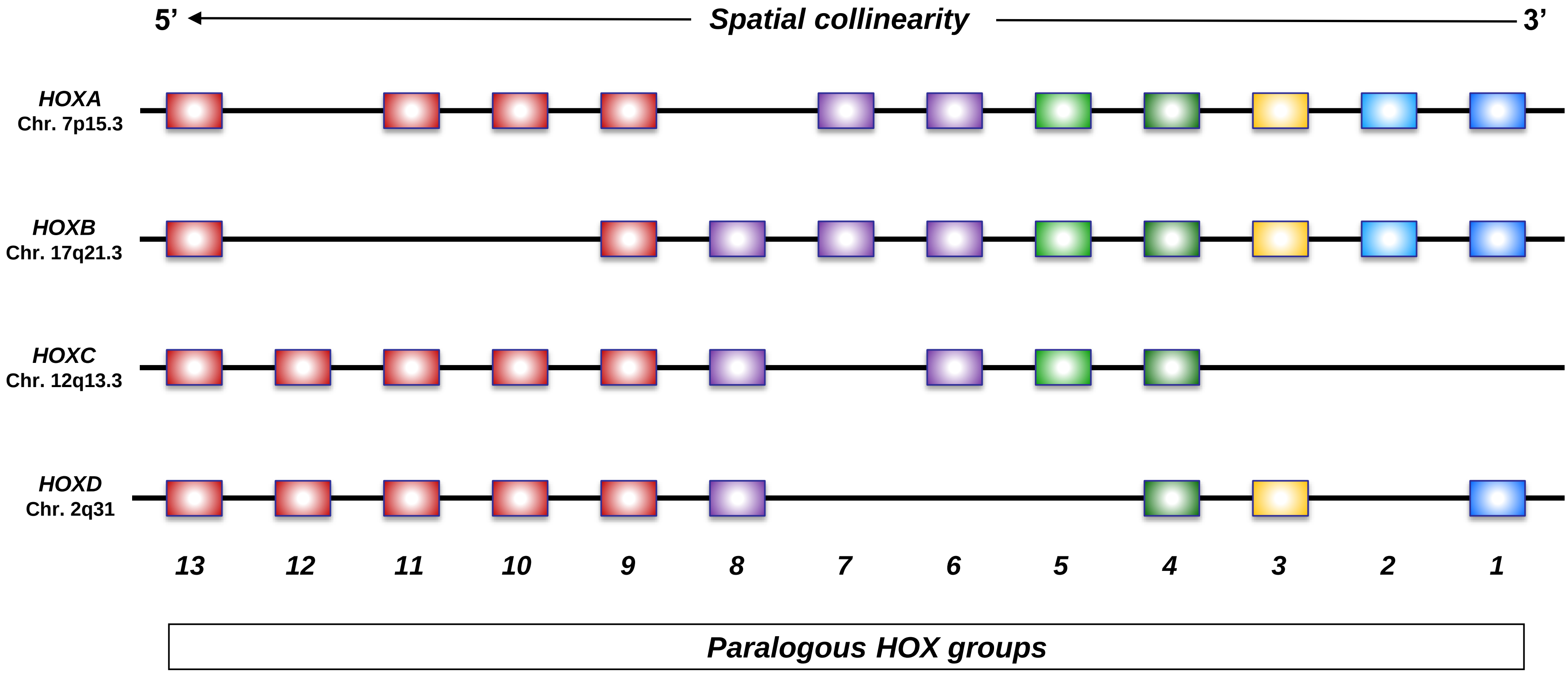

Distribution of the 39 human HOX genes in four clusters located in different chromosomal regions*. Blue indicates anterior HOX genes. Yellow, paralogy group 3 Hox genes, green and purple indicatete central HOX genes and Red the posterior HOX genes.

* Apiou F, Flagiello D, Cillo C, Malfoy B, Poupon MF, Dutrillaux B. Fine mapping of human HOX gene clusters. Cytogenet Cell Genet. 1996;73(1-2):114-5.

Supplementary file Table S3

Involvement of Homeobox genes in skeletal and other diseases.

GENE	OMIM	DISEASE ANNOTATION
Homeobox A1	601536	Athabaskan Brainstem Dysgenesis Syndrome; ABDS
Homeobox D4	142981	Homeobox D4; HOXD4
Homeobox D13	610713	Brachydactyly-Syndactyly Syndrome
Homeobox D13	192350	Vater Association
Homeobox D13	186300	Syndactyly, Type V
Homeobox D13	186000	Synpolydactyly 1; SPD1
Homeobox D13	113300	Brachydactyly, Type E1; BDE1
Homeobox D13	113200	Brachydactyly, Type D; BDD
Homeobox A13	176305	Preaxial Deficiency, Postaxial Polydactyly, And Hypospadias
Homeobox A13	140000	Hand-Foot-Uterus Syndrome
Homeobox A11	605432	Radioulnar Synostosis with Amegakaryocytic Thrombocytopenia
Homeobox D10	192950	Vertical Talus, Congenital; CVT
Homeobox D10	142984	Homeobox D10; HOXD10
Homeobox A2	612290	Microtia, Hearing Impairment, and Cleft Palate

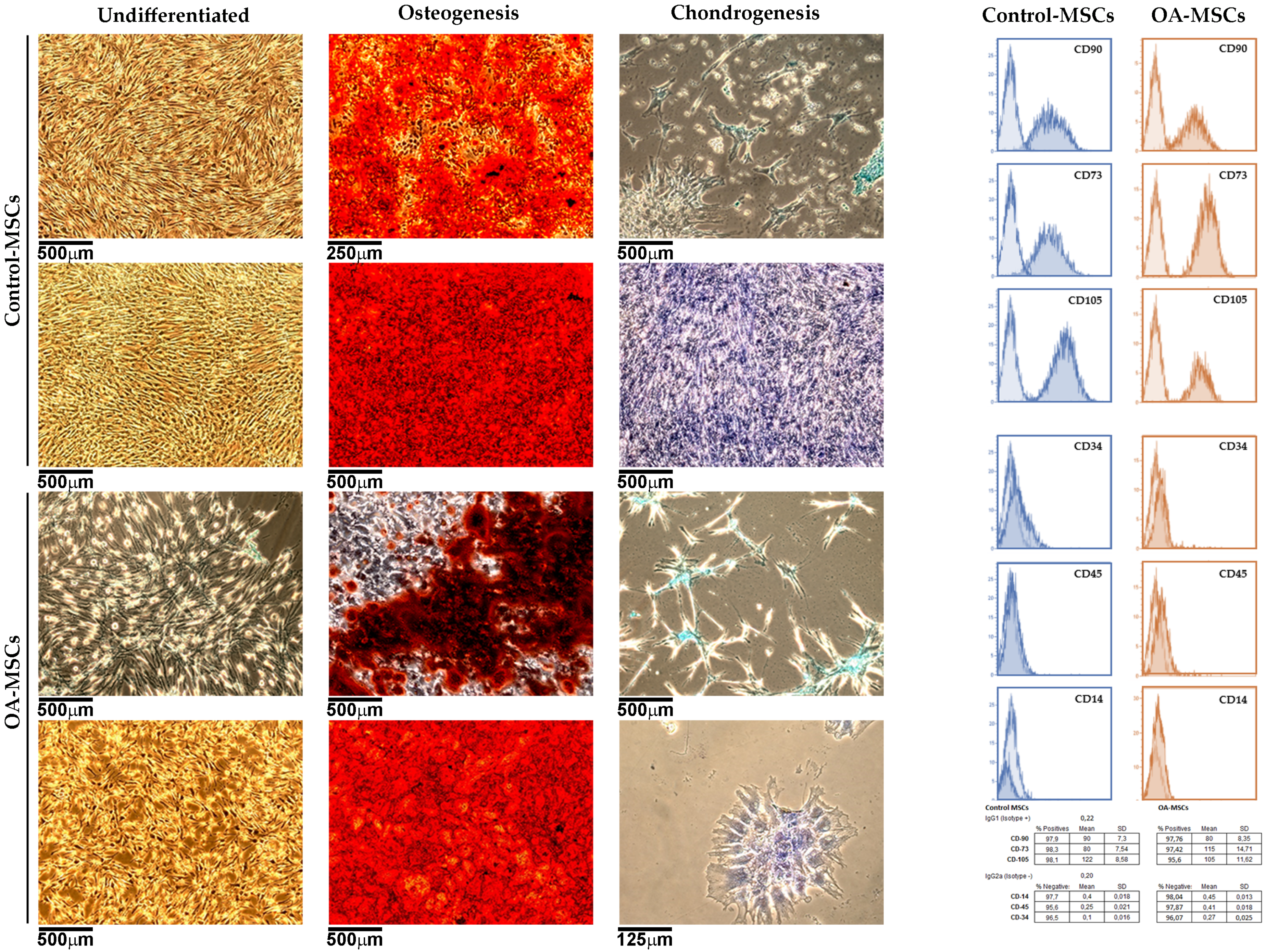

Control MSCs

IgG1 (Isotype +) 0,22

	% Positives	Mean	SD
CD-90	97,9	90	7,3
CD-73	98,3	80	7,54
CD-105	98,1	122	8,58

IgG2a (Isotype -) 0,20

	% Negative:	Mean	SD
CD-14	97,7	0,4	0,018
CD-45	95,6	0,25	0,021
CD-34	96,5	0,1	0,016

OA-MSCs

	% Positives	Mean	SD
CD-90	97,76	80	8,35
CD-73	97,42	115	14,71
CD-105	95,6	105	11,62

	% Negative:	Mean	SD
CD-14	98,04	0,45	0,013
CD-45	97,87	0,41	0,018
CD-34	96,07	0,27	0,025

Supplementary file Table S5

List of TaqMan Gene Expression Assays used in the study.

Applied Biosystems™ TaqMan® Gene Expression Assays consist of a pair of unlabeled PCR primers and a TaqMan® probe with Applied Biosystems™ FAM™ dye label on the 5′ end and minor groove binder (MGB) and nonfluorescent quencher (NFQ) on the 3′ end. **Note about HOXC12** TaqMan® Gene Expression Assays. According to Applied Biosystems and based on re-evaluation, this assay may detect genomic DNA. Housekeeping genes are indicated in red.

Gene	Catalog #	Assay ID
HOXA1	4453320	Hs00939046_m1
HOXA2	4453320	Hs00534579_m1
HOXA3	4448892	Hs00601076_m1
HOXA4	4448892	Hs01573270_m1
HOXA5	4453320	Hs00430330_m1
HOXA6	4448892	Hs00430615_m1
HOXA7	4448892	Hs00600844_m1
HOXA9	4448892	Hs04931836_s1
HOXA10	4448892	Hs00172012_m1
HOXA11	4448892	Hs00194149_m1
HOXA13	4448892	Hs00426284_m1
HOXB1	4453320	Hs00157973_m1
HOXB2	4448892	Hs01911167_s1
HOXB3	4448892	Hs01587922_m1
HOXB4	4453320	Hs00256884_m1
HOXB5	4448892	Hs00357820_m1
HOXB6	4448892	Hs00980016_m1
HOXB7	4448892	Hs04187556_m1
HOXB8	4448892	Hs00256885_m1
HOXB9	4448892	Hs00256886_m1
HOXB13	4448892	Hs00197189_m1
HOXC4	4448892	Hs00538088_m1
HOXC5	4448892	Hs00232747_m1
HOXC6	4448892	Hs00171690_m1
HOXC8	4448892	Hs00224073_m1
HOXC9	4448892	Hs00396786_m1
HOXC10	4448892	Hs00213579_m1
HOXC11	4448892	Hs00204415_m1
~~***HOXC12***~~	~~4426961~~	~~Hs00329652_s1~~
HOXC13	4448892	Hs00600868_m1
HOXD1	4448892	Hs00707081_s1
HOXD3	4448892	Hs00232506_m1
HOXD4	4448892	Hs00429605_m1
HOXD8	4448892	Hs00251905_m1
HOXD9	4448892	Hs00610725_g1
HOXD10	4453320	Hs00157974_m1
HOXD11	4448892	Hs00360798_m1
HOXD12	4448892	Hs00706957_s1
HOXD13	4448892	Hs00171253_m1
ACTB	4448892	Hs01060665_g1
18S	4453320	Hs03003631_g1
GAPDH	4331182	Hs02786624_g1

Supplementary file Table S6

TOP 100 Upregulated genes in OA-MSCs

NAME	DESCRIPTION
OGN	Osteoglycin
CHI3L2	Chitinase 3-like 2
CCRL1	Chemokine (C-C motif) receptor-like 1
CHI3L1	Chitinase 3-like 1 (cartilage glycoprotein-39)
MYOC	Myocilin, trabecular meshwork inducible glucocorticoid response
ANGPTL5	Angiopoietin-like 5
CD70	CD70 molecule
RSPO2	R-spondin 2 homolog (Xenopus laevis)
PDPN	Podoplanin
FGF18	Fibroblast growth factor 18
BARX1	BARX homeobox 1
EPB41L3	Erythrocyte membrane protein band 4.1-like 3
LNX1	Ligand of numb-protein X 1
NTN1	Netrin 1
ADH1A	Alcohol dehydrogenase 1A (class I), alpha polypeptide
MEOX2	Mesenchyme homeobox 2
MYRIP	Myosin VIIA and Rab interacting protein
MIA	Melanoma inhibitory activity
HAPLN1	Hyaluronan and proteoglycan link protein 1
PRG4	Proteoglycan 4
FGL2	Fibrinogen-like 2
SCARA5	Scavenger receptor class A, member 5 (putative)
MMP1	Matrix metallopeptidase 1 (interstitial collagenase)
FLJ14167	Hypothetical protein FLJ14167
CRTAC1	Cartilage acidic protein 1
SEMA3E	Sema domain, immunoglobulin domain (Ig), short basic domain, secreted, (semaphorin) 3E
PLA2G2A	Phospholipase A2, group IIA (platelets, synovial fluid)
RGMA	RGM domain family, member A
ADAMTSL3	ADAMTS-like 3
VEPH1	Ventricular zone expressed PH domain homolog 1 (zebrafish)
IL13RA2	Interleukin 13 receptor, alpha 2
ADH1C	Alcohol dehydrogenase 1C (class I), gamma polypeptide
FGD4	FYVE, rhogef and PH domain containing 4
ERG	V-ets erythroblastosis virus E26 oncogene homolog (avian)
COLEC12	Collectin sub-family member 12
CFB	Complement factor B
ZNF521	Zinc finger protein 521
TCEAL7	Transcription elongation factor A (SII)-like 7
CP	Ceruloplasmin (ferroxidase)
ADORA1	Adenosine A1 receptor
LAMA2	Laminin, alpha 2 (merosin, congenital muscular dystrophy)
ZNF659	Zinc finger protein 659
ZNF533	Zinc finger protein 533
CRYM	Crystallin, mu
ABCA6	ATP-binding cassette, sub-family A (ABC1), member 6
HYAL1	Hyaluronoglucosaminidase 1
FGF9	Fibroblast growth factor 9 (glia-activating factor)
KIAA1199	Kiaa1199
CDCA7L	Cell division cycle associated 7-like
CIT	Citron (rho-interacting, serine/threonine kinase 21)
OAF	OAF homolog (Drosophila)
DLX4	Distal-less homeobox 4
OMD	Osteomodulin

CPXM2	Carboxypeptidase X (M14 family), member 2
WISP3	WNT1 inducible signaling pathway protein 3
C2orf40	Chromosome 2 open reading frame 40
DLK1	Delta-like 1 homolog (Drosophila)
ADRA2A	Adrenergic, alpha-2A-, receptor
ASPN	Asporin
SCARA3	Scavenger receptor class A, member 3
DNM1	Dynamin 1
CFI	Complement factor I
EGR3	Early growth response 3
BEGAIN	Brain-enriched guanylate kinase-associated homolog (rat)
BDKRB1	Bradykinin receptor B1
DENND2A	DENN/MADD domain containing 2A
FLJ30901	Hypothetical protein FLJ30901
PCOLCE2	Procollagen C-endopeptidase enhancer 2
RIPK3	Receptor-interacting serine-threonine kinase 3
GALNT12	UDP-N-acetyl-alpha-D-galactosamine:polypeptide N-acetylgalactosaminyltransferase 12 (galnac-T12)
TEK	TEK tyrosine kinase, endothelial (venous malformations, multiple cutaneous and mucosal)
CPT1C	Carnitine palmitoyltransferase 1C
LHX2	LIM homeobox 2
LOC91461	Hypothetical protein BC007901
NNAT	Neuronatin
SNED1	Sushi, nidogen and EGF-like domains 1
DPP4	Dipeptidyl-peptidase 4 (CD26, adenosine deaminase complexing protein 2)
RBPMS2	RNA binding protein with multiple splicing 2
SECTM1	Secreted and transmembrane 1
KIAA1324L	KIAA1324-like
C10orf90	Chromosome 10 open reading frame 90
SERPINA1	Serpin peptidase inhibitor, clade A (alpha-1 antiproteinase, antitrypsin), member 1
AEBP1	AE binding protein 1
CDKN3	Cyclin-dependent kinase inhibitor 3 (CDK2-associated dual specificity phosphatase)
GPX3	Glutathione peroxidase 3 (plasma)
RARRES1	Retinoic acid receptor responder (tazarotene induced) 1
SLC47A1	Solute carrier family 47, member 1
CSDC2	Cold shock domain containing C2, RNA binding
CYP3A5	Cytochrome P450, family 3, subfamily A, polypeptide 5
CLIC2	Chloride intracellular channel 2
PAQR9	Progestin and adipoq receptor family member IX
NTF3	Neurotrophin 3
SOX5	SRY (sex determining region Y)-box 5
OSAP	Ovary-specific acidic protein
C3	Complement component 3
ITGB8	Integrin, beta 8
TNFRSF1B	Tumor necrosis factor receptor superfamily, member 1B
ASPM	Asp (abnormal spindle) homolog, microcephaly associated (Drosophila)
PITX1	Paired-like homeodomain 1

TOP 100 Downregulated genes in OA-MSCs

NAME	DESCRIPTION
IGHA2	Immunoglobulin heavy constant alpha 2 (A2m marker)
LPL	Lipoprotein lipase
IGL@	Immunoglobulin lambda locus
IGHG4	Immunoglobulin heavy constant gamma 4 (G4m marker)
IGHM	Immunoglobulin heavy constant mu
HEYL	Hairy/enhancer-of-split related with YRPW motif-like
SPP1	Secreted phosphoprotein 1 (osteopontin, bone sialoprotein I, early T-lymphocyte activation 1)
CD163	CD163 molecule
IGLL1	Immunoglobulin lambda-like polypeptide 1
CCL3	Chemokine (C-C motif) ligand 3
NTN2L	Netrin 2-like (chicken)
IGH@	Immunoglobulin heavy locus
CTA-246H3.1	Similar to omega protein
SFRP2	Secreted frizzled-related protein 2
IGLV2-14	Immunoglobulin lambda variable 2-14
TYROBP	TYRO protein tyrosine kinase binding protein
HOXC13	**Homeobox C13**
C1QB	Complement component 1, q subcomponent, B chain
PRL	Prolactin
HOXB6	**Homeobox B6**
PLA2G7	Phospholipase A2, group VII (platelet-activating factor acetylhydrolase, plasma)
CHRDL2	Chordin-like 2
WFDC1	WAP four-disulfide core domain 1
ACP5	Acid phosphatase 5, tartrate resistant
MYH11	Myosin, heavy chain 11, smooth muscle
MCAM	Melanoma cell adhesion molecule
FCGR3A	Fc fragment of igg, low affinity iiia, receptor (CD16a)
IGKV1D-13	Immunoglobulin kappa variable 1D-13
AIF1	Allograft inflammatory factor 1
FRAS1	Fraser syndrome 1
LCP1	Lymphocyte cytosolic protein 1 (L-plastin)
MS4A7	Membrane-spanning 4-domains, subfamily A, member 7
C1QA	Complement component 1, q subcomponent, A chain
IGHG1	Immunoglobulin heavy constant gamma 1 (G1m marker)
TREM2	Triggering receptor expressed on myeloid cells 2
ZMAT4	Zinc finger, matrin type 4
TM4SF20	Transmembrane 4 L six family member 20
MMP9	Matrix metallopeptidase 9 (gelatinase B, 92kda gelatinase, 92kda type IV collagenase)
SLAMF7	SLAM family member 7
COL10A1	Collagen, type X, alpha 1(Schmid metaphyseal chondrodysplasia)
IGKV1-5	Immunoglobulin kappa variable 1-5
CD52	CD52 molecule
CSF1R	Colony stimulating factor 1 receptor
FPR1	Formyl peptide receptor 1
CDH15	Cadherin 15, M-cadherin (myotubule)
ALPL	Alkaline phosphatase, liver/bone/kidney
CCL18	Chemokine (C-C motif) ligand 18 (pulmonary and activation-regulated)
PPP1R14A	Protein phosphatase 1, regulatory (inhibitor) subunit 14A
CCL3L3	Chemokine (C-C motif) ligand 3-like 3
NEFM	Neurofilament, medium polypeptide 150kda
CD53	CD53 molecule
TSPAN7	Tetraspanin 7
HEY2	Hairy/enhancer-of-split related with YRPW motif 2
FMO3	Flavin containing monooxygenase 3
DYSF	Dysferlin, limb girdle muscular dystrophy 2B (autosomal recessive)
LAIR1	Leukocyte-associated immunoglobulin-like receptor 1
TSPAN18	Tetraspanin 18

UNC5C	Unc-5 homolog C (C. Elegans)
HAS1	Hyaluronan synthase 1
HOXB4	**Homeobox B4**
LAPTM5	Lysosomal associated multispanning membrane protein 5
VMO1	Vitelline membrane outer layer 1 homolog (chicken)
CXCR4	Chemokine (C-X-C motif) receptor 4
CNN1	Calponin 1, basic, smooth muscle
LOC401233	Similar to HIV TAT specific factor 1; cofactor required for Tat activation of HIV-1 transcription
HOXB3	**Homeobox B3**
HCLS1	Hematopoietic cell-specific Lyn substrate 1
JPH2	Junctophilin 2
EPC1	Enhancer of polycomb homolog 1 (Drosophila)
COBL	Cordon-bleu homolog (mouse)
IGKC	Immunoglobulin kappa constant
COL11A1	Collagen, type XI, alpha 1
SLC16A6	Solute carrier family 16, member 6 (monocarboxylic acid transporter 7)
BEX1	Brain expressed, X-linked 1
IGHV4-31	Immunoglobulin heavy variable 4-31
MGAT4A	Mannosyl (alpha-1,3-)-glycoprotein beta-1,4-N-acetylglucosaminyltransferase, isozyme A
FLRT3	Fibronectin leucine rich transmembrane protein 3
IGJ	Immunoglobulin J polypeptide, linker protein for immunoglobulin alpha and mu polypeptides
KIF26B	Kinesin family member 26B
PLCG2	Phospholipase C, gamma 2 (phosphatidylinositol-specific)
MS4A4A	Membrane-spanning 4-domains, subfamily A, member 4
C15orf48	Chromosome 15 open reading frame 48
NR4A3	Nuclear receptor subfamily 4, group A, member 3
ASB2	Ankyrin repeat and SOCS box-containing 2
RNF128	Ring finger protein 128
KCNMB4	Potassium large conductance calcium-activated channel, subfamily M, beta member 4
KRT7	Keratin 7
NGFR	Nerve growth factor receptor (TNFR superfamily, member 16)
MGC23985	Similar to AVLV472
CCL4	Chemokine (C-C motif) ligand 4
CES1	Carboxylesterase 1 (monocyte/macrophage serine esterase 1)
LILRB3	Leukocyte immunoglobulin-like receptor, subfamily B (with TM and ITIM domains), member 3
PLN	Phospholamban
ADAMTS9	ADAM metallopeptidase with thrombospondin type 1 motif, 9
HS3ST2	Heparan sulfate (glucosamine) 3-O-sulfotransferase 2
OASL	2'-5'-oligoadenylate synthetase-like
CDH3	Cadherin 3, type 1, P-cadherin (placental)
HLA-DMB	Major histocompatibility complex, class II, DM beta
ANKRD1	Ankyrin repeat domain 1 (cardiac muscle)
SIX6	SIX homeobox 6

Top 100 upregulated and downregulated genes in OA-MSCs from the DNA-Array. In red are indicated all the homeobox genes and **Hox clustered** genes are in bold.

Supplementary file Table S7

Enrichment in OA phenotype (8 samples)

2 / 4 gene sets are **upregulated** in phenotype OA: HOXA and HOXD
Only 1 gene set (HOMEOBOXA) is significant at FDR < 25%

NAME	SIZE	NOM p-val	FDR q-val	FWER p-val
HOMEOBOX A	6	0.00193	0.00303	0.003

PROBE	DESCRIPTION (from dataset)	GENE SYMBOL	RANK IN GENE LIST	CORE ENRICHMENT
HOXA13	**homeobox A13**	***HOXA13***	**380**	**Yes**
HOXA5	**homeobox A5**	***HOXA5***	**1080**	**Yes**
HOXA9	**homeobox A9**	***HOXA9***	**1382**	**Yes**
HOXA7	**homeobox A7**	***HOXA7***	**1668**	**Yes**
HOXA11	**homeobox A11**	***HOXA11***	**1987**	**Yes**
HOXA10	**homeobox A10**	***HOXA10***	**3486**	**Yes**
HOXA3	homeobox A3	*HOXA3*	7346	No
HOXA4	homeobox A4	*HOXA4*	13707	No
HOXA2	homeobox A2	*HOXA2*	13778	No

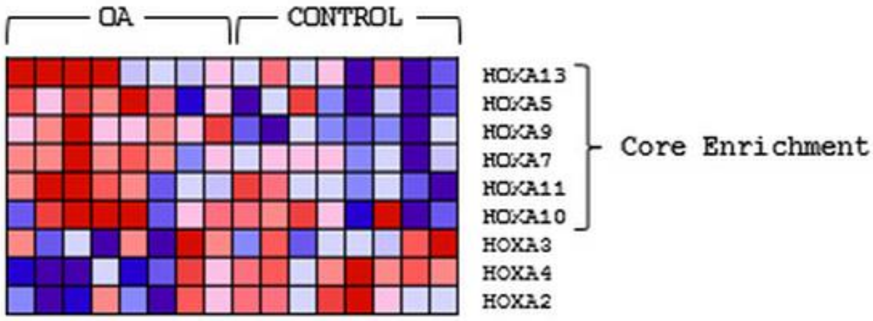

PROBE	DESCRIPTION (from dataset)	GENE SYMBOL	RANK IN GENE LIST	CORE ENRICHMENT
HOXD3	**homeobox D3**	***HOXD3***	**380**	**Yes**
HOXD8	**homeobox D8**	***HOXD8***	**1080**	**Yes**
HOXD9	**homeobox D9**	***HOXD9***	**1382**	**Yes**

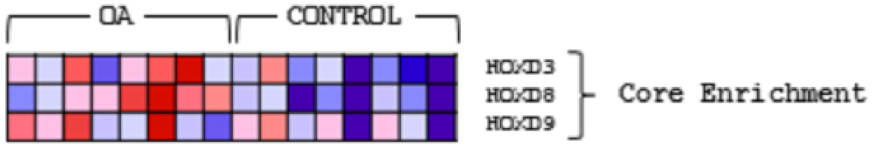

Enrichment in phenotype: CONTROL (8 samples)

2 / 4 gene sets are upregulated in phenotype CONTROL
2 gene sets are significantly enriched at FDR < 25%
1 gene sets are significantly enriched at nominal pvalue < 1%
1 gene sets are significantly enriched at nominal pvalue < 5%

NAME	SIZE	NOM p-val	FDR q-val	FWER p-val
HOMEOBOX B	6	0.00193	0.00303	0.003
HOMEOBOX C	7	0.11666	0.11344	0.172

1 gene set (HOMEOBOX B) is significantly enriched at nominal pvalue < 1%

PROBE	DESCRIPTION (from dataset)	GENE SYMBOL	RANK IN GENE LIST	CORE ENRICHMENT
HOXB7	homeobox B7	HOXB7	6427	No
HOXB2	homeobox B2	HOXB2	13273	Yes
**HOXB13**	**homeobox B13**	**HOXB13**	**13836**	**Yes**
**HOXB3**	**homeobox B3**	**HOXB3**	**14288**	**Yes**
**HOXB4**	**homeobox B4**	**HOXB4**	**14294**	**Yes**
**HOXB6**	**homeobox B6**	**HOXB6**	**14334**	**Yes**

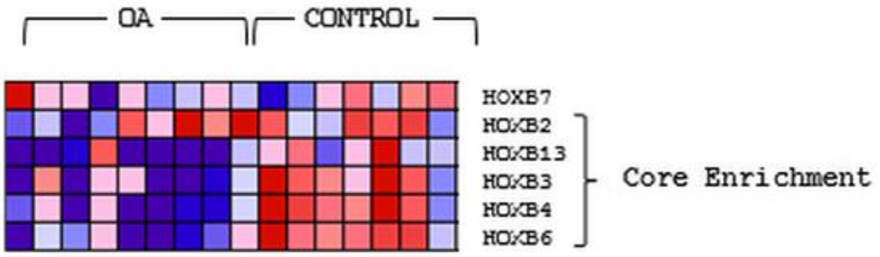

1 gene set (HOMEOBOX C) is significantly enriched at nominal pvalue < 25%

PROBE	DESCRIPTION (from dataset)	GENE SYMBOL	RANK IN GENE LIST	CORE ENRICHMENT
HOXC6	homeobox C6	HOXC6	3919	No
HOXC5	homeobox C5	HOXC5	6455	No
HOXC10	homeobox C10	HOXC10	8916	No
**HOXC9**	**homeobox C9**	**HOXC9**	**12460**	**Yes**
**HOXC8**	**homeobox C8**	**HOXC8**	**13511**	**Yes**
**HOXC12**	**homeobox C12**	**HOXC12**	**14209**	**Yes**
**HOXC13**	**homeobox C13**	**HOXC13**	**14337**	**Yes**

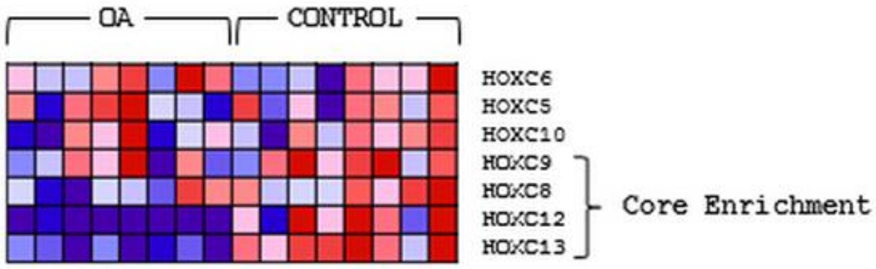

Supplementary file Table S8

HOX gene functional signatures are enriched in bone marrow Mesenchymal Stem Cells

Enrichment in OA subjects (8 samples)

5 / 12 gene sets are upregulated in OA phenotype

2 gene sets are significant at FDR < 25%
0 gene sets are significantly enriched at nominal pvalue < 1%
0 gene sets are significantly enriched at nominal pvalue < 5%

GENE SET NAME	SIZE	NOM p-val	FDR q-val
HOXA	**9**	**0.0613**	**0.2324**
**Other HOX Genes**	**5**	**0.1232**	**0.2183**
Body Pattern Formation	10	0.1080	0.2963
Anatomical Structure Morphogenesis	5	0.2202	0.2788
Hox Genes Involved In Cell Differentiation	7	0.4541	0.4401

Enrichment in control subjects (8 samples)

7 / 12 gene sets are upregulated in phenotype CONTROL

6 gene sets are significantly enriched at FDR < 25%
1 gene sets are significantly enriched at nominal pvalue < 1%
3 gene sets are significantly enriched at nominal pvalue < 5%

GENE SET NAME	SIZE	NOM p-val	FDR q-val
**Other Hox Involved Multicellula Rorganismal Dev.**	**17**	**0.004**	**0.029**
HOXC	**6**	**0.035**	**0.128**
**Multicellular Organismal development (Hox)**	**7**	**0.035**	**0.094**
**Body Pattern Formation (Hox)**	**5**	**0.066**	**0.115**
**Skeletal Development**	**10**	**0.151**	**0.206**
**Nervous System Development**	**5**	**0.232**	**0.247**
**Organ Morphogenesis**	7	0.979	0.988

HOXA

HOXA13	homeobox A13
HOXA5	homeobox A5
HOXA9	homeobox A9
HOXA7	homeobox A7
HOXA11	homeobox A11
HOXA10	homeobox A10
HOXA3	homeobox A3
HOXA4	homeobox A4
HOXA2	homeobox A2

BODY PATTERN FORMATION

EMX2	empty spiracles homeobox 2
HHEX	hematopoietically expressed homeobox
HOXA5	homeobox A5
HOXD8	homeobox D8
HOXA11	homeobox A11
HOXC5	homeobox C5
PITX2	paired-like homeodomain 2
HOXA4	homeobox A4
HOXA2	homeobox A2
HOXB6	homeobox B6

OTHER GENES

SIRT3	sirtuin (silent mating type information regulation 2 homolog) 3 (S. cerevisiae)
SIRT7	sirtuin (silent mating type information regulation 2 homolog) 7 (S. cerevisiae)
ZHX2	zinc fingers and homeoboxes 2
SIRT6	sirtuin (silent mating type information regulation 2 homolog) 6 (S. cerevisiae)
ZHX1	zinc fingers and homeoboxes 1

ANATOMICAL STRUCTURE MORPHOGENESIS

HOXD3	homeobox D3
SIX4	SIX homeobox 4
SIX2	SIX homeobox 2
LBX1	ladybird homeobox 1
HOXC13	homeobox C13

HOX GENES INVOLVED IN CELL DIFFERENTIATION

EMX2	empty spiracles homeobox 2
HHEX	hematopoietically expressed homeobox
LHX1	LIM homeobox 1
LBX1	ladybird homeobox 1
SIRT1	sirtuin (silent mating type information regulation 2 homolog) 1 (S. cerevisiae)
PHOX2A	paired-like homeobox 2a
HLX	H2.0-like homeobox

OTHER HOMEOBOX GENES INVOLVED IN MULTICELLULAR ORGANISMAL DEVELOPMENT

HOXA9	homeobox A9
HOXD9	homeobox D9
HOXA7	homeobox A7
HOXB7	homeobox B7
EN2	engrailed homeobox 2

EMX1	empty spiracles homeobox 1
LBX2	ladybird homeobox 2
DLX1	distal-less homeobox 1
HOXC9	homeobox C9
MEIS1	Meis homeobox 1
HOXB2	homeobox B2
HOXC8	homeobox C8
NKX3-1	NK3 homeobox 1
HOXB13	homeobox B13
HOXC12	homeobox C12
HOXB3	homeobox B3
HOXB4	homeobox B4

MULTICELLULAR ORGANISMAL DEVELOPMENT(HOX)

HOXA9	homeobox A9
HOXA7	homeobox A7
HOXB7	homeobox B7
HOXB2	homeobox B2
HOXC8	homeobox C8
HOXB13	homeobox B13
HOXB4	homeobox B4

HOXC

HOXC6	homeobox C6
HOXC5	homeobox C5
HOXC9	homeobox C9
HOXC8	homeobox C8
HOXC12	homeobox C12
HOXC13	homeobox C13

BODY PATTERN FORMATION (HOX)

HOXD3	homeobox D3
HOXA5	homeobox A5
HOXA4	homeobox A4
HOXA2	homeobox A2
HOXB6	homeobox B6

SKELETAL DEVELOPMENT

HOXA13	homeobox A13
DLX3	distal-less homeobox 3
MSX2	msh homeobox 2
HOXA11	homeobox A11
EN1	engrailed homeobox 1
ALX4	aristaless-like homeobox 4
DLX6	distal-less homeobox 6
HOXA2	homeobox A2
DLX5	distal-less homeobox 5
HOXB6	homeobox B6

NERVOUS SYSTEM DEVELOPMENT

LHX1	LIM homeobox 1
LBX1	ladybird homeobox 1
PHOX2A	paired-like homeobox 2a

DLX6	distal-less homeobox 6
DLX5	distal-less homeobox 5

ORGAN MORPHOGENESIS

HOXA13	homeobox A13
HOXA11	homeobox A11
LHX1	LIM homeobox 1
PITX3	paired-like homeodomain 3
PITX2	paired-like homeodomain 2
PDHX	pyruvate dehydrogenase complex, component X
SIX6	SIX homeobox 6

Supplementary file Table S9

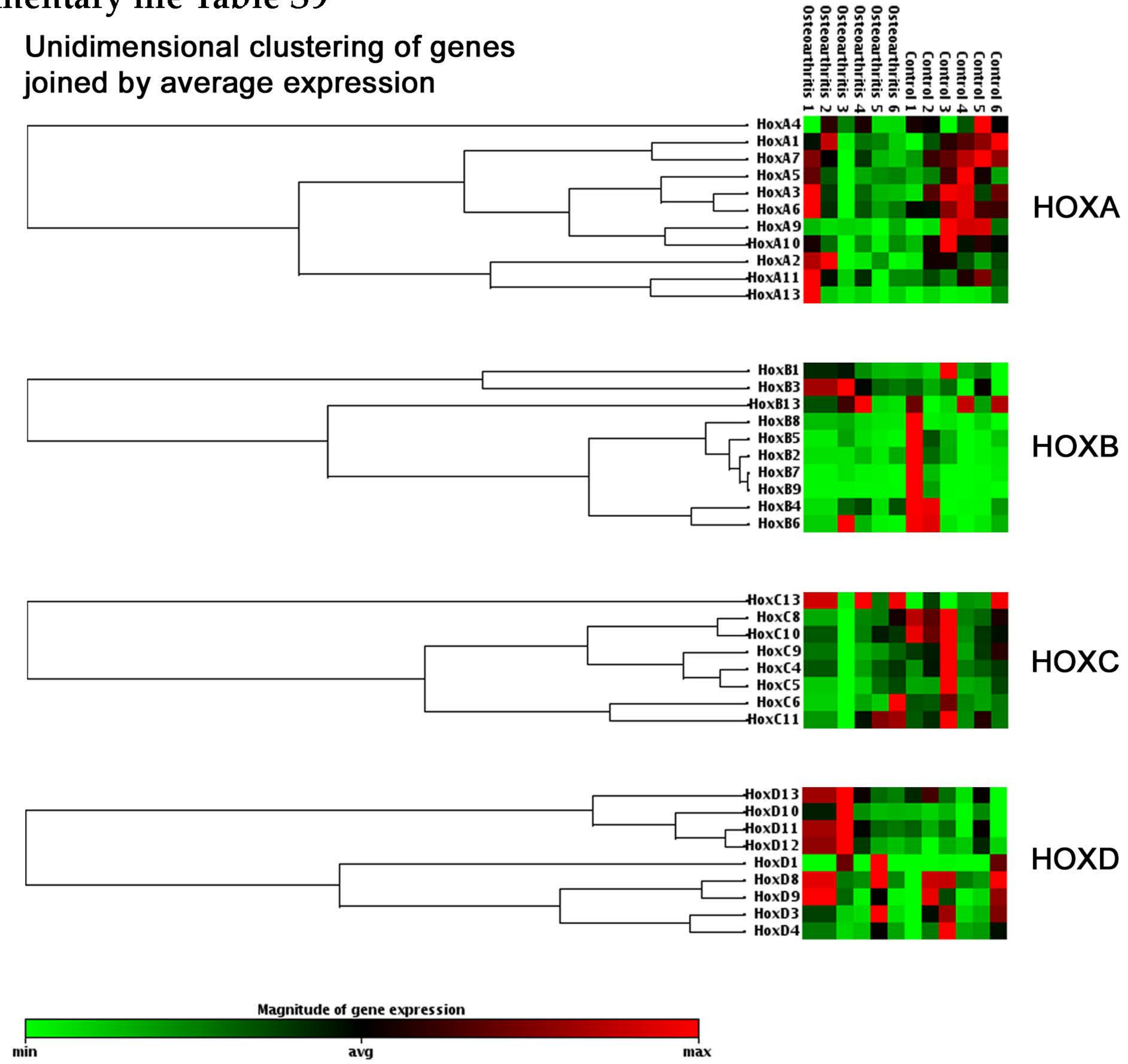

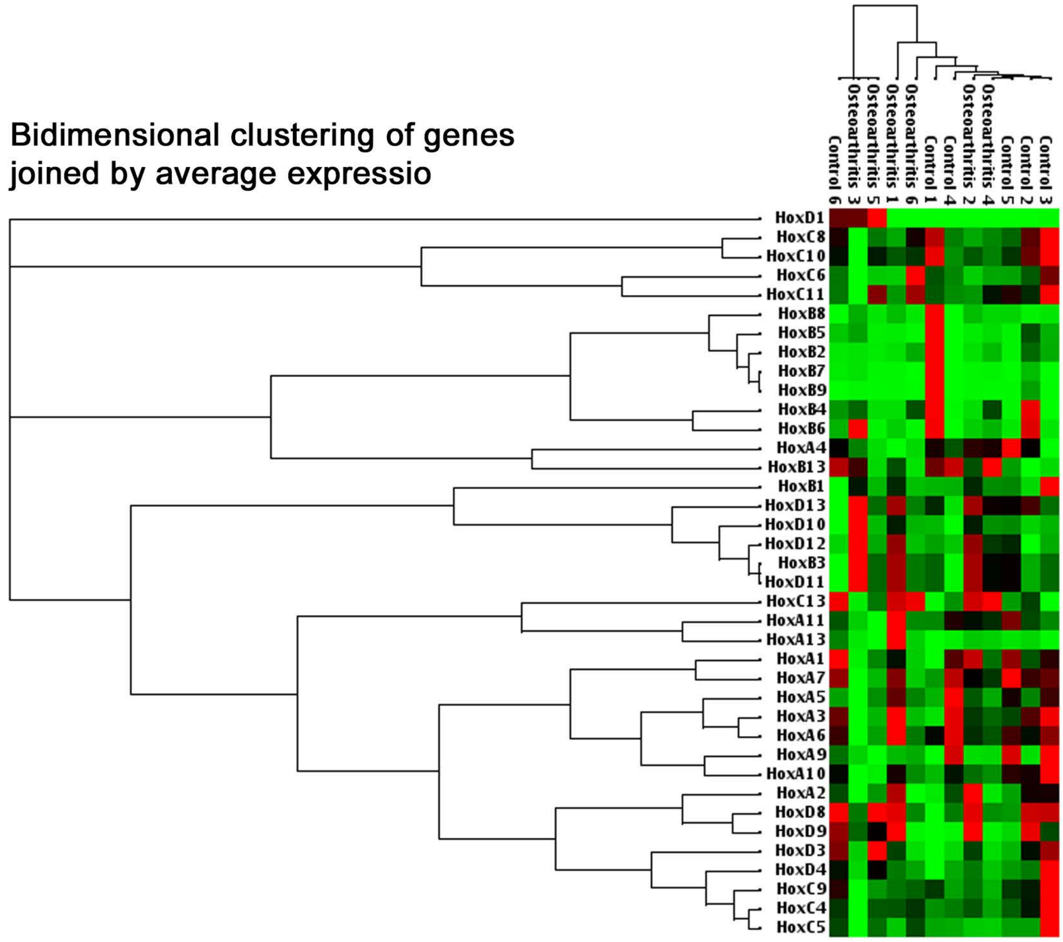

Supplement: Supplementary file 1 [file cells-07-00244-s001.pdf]
